# Investigating split‐filter dual‐energy CT for improving liver tumor visibility for radiation therapy

**DOI:** 10.1002/acm2.12904

**Published:** 2020-05-15

**Authors:** Lianna D. DiMaso, Jessica R. Miller, Michael J. Lawless, Michael F. Bassetti, Larry A. DeWerd, Jessie Huang

**Affiliations:** ^1^ Department of Medical Physics University of Wisconsin‐Madison Madison WI USA; ^2^ Department of Human Oncology University of Wisconsin‐Madison Madison WI USA

**Keywords:** dual‐energy, liver tumor, split‐filter, TwinBeam, visibility

## Abstract

**Purpose:**

Accurate liver tumor delineation is crucial for radiation therapy, but liver tumor volumes are difficult to visualize with conventional single‐energy CT. This work investigates the use of split‐filter dual‐energy CT (DECT) for liver tumor visibility by quantifying contrast and contrast‐to‐noise ratio (CNR).

**Methods:**

Split‐filter DECT contrast‐enhanced scans of 20 liver tumors including cholangiocarcinomas, hepatocellular carcinomas, and liver metastases were acquired. Analysis was performed on the arterial and venous phases of mixed 120 kVp‐equivalent images and VMIs at 57 keV and 40 keV gross target volume (GTV) contrast and CNR were calculated.

**Results:**

For the arterial phase, liver GTV contrast was 12.1 ± 10.0 HU and 43.1 ± 32.3 HU (*P* < 0.001) for the mixed images and 40 keV VMIs. Image noise increased on average by 116% for the 40 keV VMIs compared to the mixed images. The average CNR did not change significantly (1.6 ± 1.5, 1.7 ± 1.4, 2.4 ± 1.7 for the mixed, 57 keV and 40 keV VMIs (*P *> 0.141)). For individual cases, however, CNR increases of up to 607% were measured for the 40 keV VMIs compared to the mixed image. Venous phase 40 keV VMIs demonstrated an average increase of 35.4 HU in GTV contrast and 121% increase in image noise. Average CNR values were also not statistically different, but for individual cases CNR increases of up to 554% were measured for the 40 keV VMIs compared to the mixed image.

**Conclusions:**

Liver tumor contrast was significantly improved using split‐filter DECT 40 keV VMIs compared to mixed images. On average, there was no statistical difference in CNR between the mixed images and VMIs, but for individual cases, CNR was greatly increased for the 57 keV and 40 keV VMIs. Therefore, although not universally successful for our patient cohort, split‐filter DECT VMIs may provide substantial gains in tumor visibility of certain liver cases for radiation therapy treatment planning.

## 1 INTRODUCTION

Liver cancer is one of the leading causes of cancer‐related deaths in the United States. Unfortunately, at the time of diagnosis, the majority of cases are advanced and therefore not candidates for curative treatment. Surgical resection is the established curative treatment but because of the extent of the majority of liver tumors and their venous involvement at diagnosis, they are unresectable. Radiation therapy is the most common localized treatment option for unresectable liver cancers, and recent studies have shown that dose‐escalated stereotactic body radiotherapy (SBRT) improves local control and may decrease tumor size for resection.^1^ However, dose‐escalated SBRT requires precision targeting which can be challenging due to inaccurate liver tumor delineation on conventional computed tomography (CT) images.^1^ Several studies have demonstrated that dual‐energy CT (DECT) greatly improves the delineation and conspicuity of liver tumors.^2^, ^3^

Dual‐energy CT is the acquisition of two 3‐dimensional attenuation datasets using both low‐ and high‐energy photon spectra during a single CT protocol. The low‐ and high‐energy spectra are commonly achieved through fast kVp switching, two sequential scans, dual‐layer detector, or using two x‐ray sources. The low‐ and high‐energy spectra are also achievable by placing filters within the beam to alter the mean energy of the spectra. DECT allows for the differentiation of tissues with the same density but different elemental composition and therefore has significant advantages over conventional SECT, specifically when imaging the abdomen.^2^ When imaging the liver, low‐energy images created from sequential scanning and fast kVp‐switching DECT have been shown to increase iodine conspicuity and increase contrast of hypervascular liver tumors, including hepatocellular carcinomas (HCC) and metastases.^3^, ^4^, ^5^, ^6^

When considering the type of DECT modality, greater spectral separation results in better tissue differentiation, and greater temporal coherence between the low‐ and high‐energy acquisitions reduces the impact of artifacts for dynamic contrast imaging. In addition to the previously mentioned techniques, single‐source DECT can also be achieved using a split‐filter technique available on the Siemens SOMATOM Definition Edge CT scanner (Siemens Healthcare, Forchheim, Germany). The Edge has an acquisition technique known as TwinBeam which introduces a gold and tin split filter for DECT acquisition. TwinBeam is a cost‐effective and innovative DECT system. Although TwinBeam has a smaller time interval between the acquisition of the low‐ and high‐energy data than sequential dual‐energy scanning, it is more susceptible to artifacts when compared to fast‐kVp switching, dual‐source, and dual‐layer detector DECT. The low‐ and high‐energy datasets are acquired within two tube rotations making this modality applicable for dynamic contrast imaging. Therefore, TwinBeam may also be beneficial for abdominal cancer imaging since studies have shown that two‐phase imaging increases the detection of liver tumors.^7^ However, a disadvantage of TwinBeam is a lower spectral separation and, consequently, an inferior ability to differentiate tissues in comparison to other DECT techniques.^3^, ^8^, ^9^ DECT techniques that utilize a low‐energy 80 kVp and high‐energy 140 kVp beams have been shown to increase liver tumor detection, but there has not been any study investigating the benefits of TwinBeam DECT on liver tumor delineation for radiation therapy applications. Much like a recent study that investigated the delineation of pancreas tumors using TwinBeam, this work investigates the gross target volume (GTV) contrast and contrast‐to‐noise ratio (CNR).^10^ This work investigates several types of liver tumors, unlike recent DECT studies that have solely investigated hypo‐ or hypervascular liver tumors.^1^, ^3^, ^4^, ^6^, ^7^ Liver metastases, hepatocellular carcinomas (HCC), and cholangiocarcinomas are all included for investigation in this work. The goal of this work is to quantitatively determine if TwinBeam DECT can improve the contrast and CNR of liver tumors in comparison to conventional single‐energy CT imaging methods, with the long‐term goal of improving the delineation of these tumors for radiation therapy treatment planning purposes.

## 2 MATERIALS AND METHODS

### 2.1 Patient population and CT simulation

Patient information was collected for this study after Institutional Review Board approval for patients who received dual‐energy imaging at CT simulation for radiation therapy at our institution between June 2016 and August 2018. Of the 20 patients with liver cancer who were included in this study, 14 were men and 9 were women. The mean ± SD (range) of age was 67.1 ± 10 (39–83) years. The mean ± SD (range) of weight was 82.5 ± 12 (56.8–107.9) kg. On the basis of either histopathologic analysis or imaging follow up, 6 tumors were diagnosed as intrahepatic cholangiocarcinoma, 10 as metastatic liver cancer, and 4 as hepatocellular carcinoma. The study population included Stage I–IV liver cancer and Stage IV cancer of the esophagus, colon, and rectum that metastasized to the liver. The longest tumor dimension ranged from 1 to 14 cm.

The image acquisition has been thoroughly described in a previous study investigating pancreas tumors.^10^ For this study, all patients were imaged with a dual‐phase imaging protocol. The arterial and portal venous phase scans were acquired using patient‐specific delays based on automatic bolus tracking of the abdominal aorta. Once the iodinated contrast medium was administered, a 15 s timer initiated the monitoring of mean HU within the descending abdominal aorta. Once the threshold of 75 HU was reached, the set delay times were adjusted based on the duration of each scan so that the center of each scan was 6 and 16 s posttrigger for the arterial and portal venous phase, respectively. The effective mAs for the scans ranged between 1350 and 1500 mAs. Automatic tube current modulation was not used, and the CTDIvol ranged from 20.06 to 27.73 mGy. Images were acquired with a pitch ranging from 0.25 to 0.45, a rotation time of either 0.5 or 1 s per rotation, and reconstruction slice thickness of 3 mm. The arterial and venous phase datasets were acquired for all patients with the exception of one where only the venous phase was acquired.

Prior to radiation treatment planning, all patients were simulated on the Siemens SOMATOM Defintion Edge with TwinBeam during maximum inhalation breath hold using the Varian RPM^TM^‐guided system (Real‐time Position Management, Varian Medical Systems, Palo Alto, CA, USA) to minimize motion. Vac‐Lok^TM^ (CIVCO Radiotherapy) cushions were used as immobilization devices. All patients were scanned with a dual‐phase imaging protocol with the same amount of OMNIPAQUE™ IV nonionized iodine contrast medium regardless of patient weight. A bolus tracking technique was used to achieve the appropriate delay times.

### 2.2 Image reconstruction

**Fig. 1:**
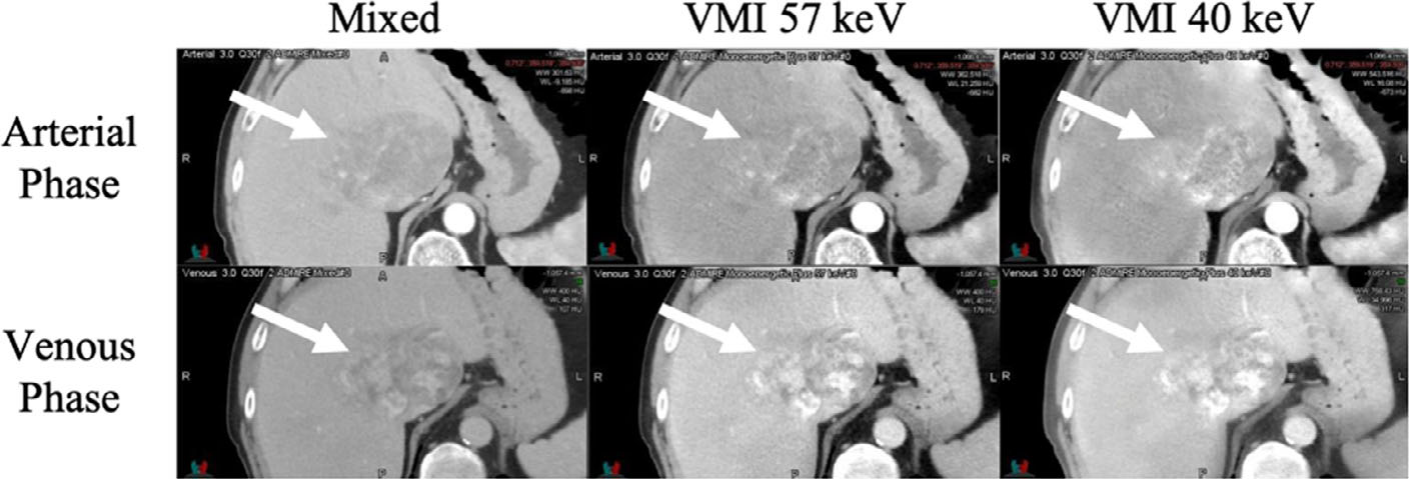
Mixed 120 kVp‐equivalent image, virtual monoenergetic image at 57 keV (VMI 57 keV), and VMI at 40 keV (VMI 40 keV) from the arterial and venous phase, illustrating the six datasets analyzed for each patient. The arrow indicates the location of the GTV.

Each raw dataset was reconstructed using the Siemens’ iterative reconstruction algorithm, ADMIRE, at a strength of 2 of 5. ADMIRE 2 was used because it has shown to decrease image noise by 20% and is preferred strength by clinicians.^10^ A mixed 120 kVp‐equivalent image, a virtual monoenergetic image (VMI) at 57 keV, and a VMI at 40 keV were then generated for each phase, for a total of 6 different image sets for each liver tumor case (Fig. 1). The VMIs at 57 keV and 40 keV were generated using the Siemens Monoenergetic + application.

### 2.3 Contrast and contrast‐to‐noise ratio

The MIMvista software (MIM Software Inc. Cleveland, OH, USA) was used to analyze each image. The entire liver GTV was segmented by an experienced radiation oncologist on the arterial phase VMI at 57 keV, similar to what is done for radiation treatment planning at our institution. To investigate liver tumor GTV contrast, the surrounding healthy liver tissue was assessed using a nearby region of interest (ROI) placed within a homogenous region of healthy liver parenchyma avoiding any vessels and bile ducts. A 10 mm^2^ ROI placed within a homogeneous region of the erector spinae muscle was used to assess image noise.

Gross target volume contrast was calculated as the absolute difference in HU between the healthy liver parenchyma and GTV, ${\text{GTV}}_{\text{contrast}}=\left|{\bar{\text{HU}}}_{\text{liver}}-{\bar{\text{HU}}}_{\text{GTV}}\right|$. GTV contrast was divided by the standard deviation of the ROI located in the erector spinae muscle to calculate GTV CNR, ${\text{GTV}}_{\text{CNR}}=\frac{\left|{\bar{\text{HU}}}_{\text{liver}}-{\bar{\text{HU}}}_{\text{GTV}}\right|}{\sigma }$. The absolute contrast difference was used to calculate GTV contrast and CNR in order to analyze hypoattenuating and hyperattenuating liver tumors using the same methodology.

MATLAB was used for all statistical analyses. The difference in absolute GTV contrast and CNR between the mixed 120 kVp‐equivalent images and VMIs was analyzed using paired t‐tests. Statistical significance was determined using a *P*‐value less than 0.05.

## 3 RESULTS

### 3.1 Absolute contrast

Table 1 lists the mean liver GTV absolute contrast from the mixed 120 kVp‐equivalent images, VMIs at 57 keV, and VMIs at 40 keV for both arterial and venous phases. The mean ± standard deviation (SD) GTV contrast for the arterial phase and venous phase from all datasets was 12.1 ± 10.0 HU and 19.5 ± 13.4 HU for the mixed 120 kVp‐equivalent images, respectively. The VMIs at 57 keV had a greater GTV contrast of 21.5 ± 15.4 HU and 30.9 ± 18.7 HU for the arterial and venous phase, respectively, representing a 77% and 58% increase (*P* = 0.04 and 0.03). The VMIs at 40 keV showed the greatest GTV contrast of 43.1 ± 32.3 HU and 54.3 ± 32.6 HU for the arterial and venous phase, respectively, which represent a 255% and 179% increase from the mixed 120 kVp‐equivalent images (*P* < 0.001). Although on average the venous phase showed the greatest GTV contrast, some cases had a greater GTV contrast during the arterial phase.

**Table 1:** Mean ± SD (Range) GTV absolute contrast, image noise, and CNR of all cases from both contrast phases with ADMIRE 2 reconstructed mixed 120 kVp‐equivalent images (Mixed), virtual monoenergetic images at 57 keV (VMI 57 keV), and at 40 keV (VMI 40 keV)

	Mixed	VMI 57 keV	VMI 40 keV	*p‐*value^a^	*p‐*value^b^	*p‐*value^c^
Contrast (HU)						
Arterial Phase + ADMIRE	12.1 ± 10.0	21.5 ± 15.4	43.1 ± 32.3	0.039	0.000	0.013
Venous Phase + ADMIRE	19.5 ± 13.4	30.9 ± 18.7	54.3 ± 32.6	0.033	0.000	0.008
Images noise (HU)						
Arterial Phase + ADMIRE	8.1 ± 1.6	12.4 ± 2.0	17.5 ± 2.7	0.000	0.000	0.004
Venous Phase + ADMIRE	8.4 ± 1.0	13.0 ± 1.5	18.5 ± 2.2	0.000	0.000	0.000
CNR						
Arterial Phase + ADMIRE	1.6 ± 1.5	1.7 ± 1.4	2.4 ± 1.7	0.798	0.131	0.141
Venous Phase + ADMIRE	2.4 ± 1.7	2.4 ± 1.5	2.9 ± 1.8	0.977	0.307	0.485

All values are giving in mean ± SD except for *p*‐values.

CNR, contrast‐to‐noise ratio.

^a^ Paired t‐test comparing columns Mixed and VMI 57 keV.

^b^ Paired t‐test comparing columns Mixed and VMI 40 keV.

^c^ Paired t‐test comparing columns VMI 57 keV and VMI 40 keV.

### 3.2 Noise

There was no statistical difference in noise between the arterial and venous phase across all images (*P> *0.05). The mean ± SD image noise of the mixed 120 kVp‐equivalent images, VMI at 57 keV, and VMIs at 40 keV was 8.1 ± 1.6 HU, 12.7 ± 2.0 HU, and 18.0 ± 2.7 HU, respectively (*P* < 0.001). Image noise was about 50% higher for the VMIs at 57 keV and 120% higher for the VMIs at 40 keV compared to the mixed 120 kVp‐equivalent images.

### 3.3 Contrast‐to‐noise ratio

The mean ± SD of GTV CNR across all tumor cases investigated are listed in Table 1. The arterial phase datasets showed an 8% and 50% increase in mean GTV CNR for the VMIs at 57 keV and 40 keV compared to the mixed 120 kVp equivalent. This gain in CNR was statistically insignificant (*P* = 0.80, *P* = 0.13). For the venous phase datasets, there was no statistical difference in GTV CNR between the mixed 120 kVp‐equivalent images and VMIs at 57 keV (2.4 ± 1.7 and 2.4 ± 1.5). There was, however, a 24% increase in GTV CNR from the mixed 120 kVp‐equivalent images to the VMIs at 40 keV (*P* = 0.31).

**Fig. 2:**
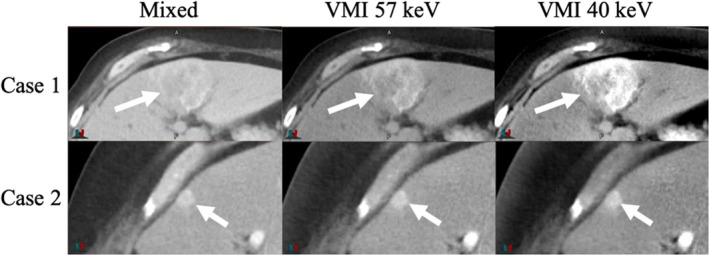
Mixed, VMI at 57 keV, and VMI at 40 keV of two tumor cases that showed the greatest tumor contrast during the arterial phase (Case 1) and during the venous phase (Case 2). The arrow indicated the location of the tumor

**Fig. 3:**
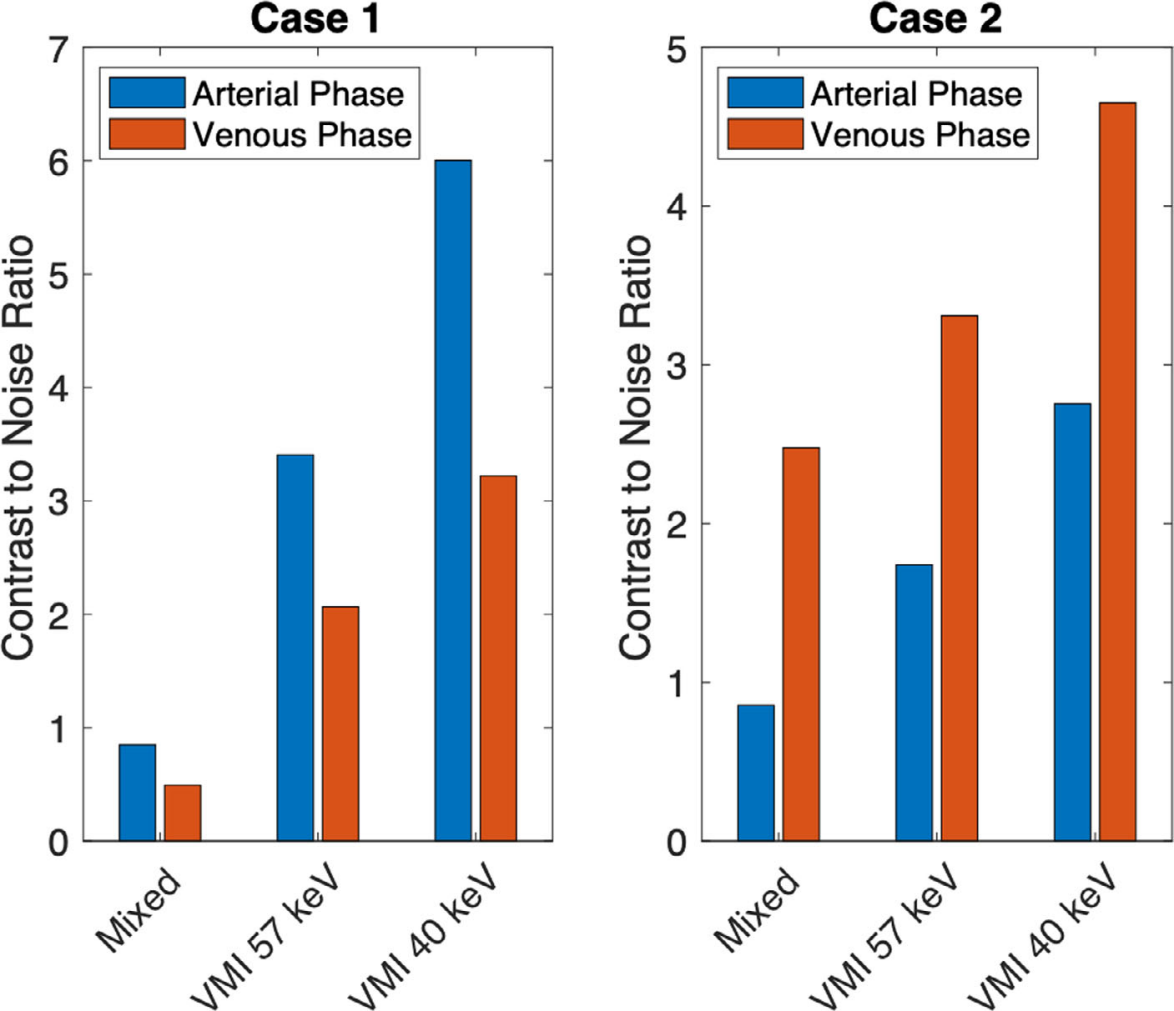
GTV CNR from Case 1 and Case 2 illustrated in Fig. 2

Although there was no statistical difference in mean GTV CNR, there were cases where the VMIs showed much greater GTV CNR compared to the mixed 120 kVp‐equivalent images. Figures 2 and 3 show two specific cases where the VMIs provided gain in GTV CNR. For the arterial phase of case 1, the GTV CNR was increased from 0.85 to 3.41 and 6.00 for VMI at 57 keV and 40 keV compared to the mixed 120 kVp‐equivalent image. For the venous phase of case 2, the GTV CNR was increased from 1.97 to 2.61 and 3.78 for VMI at 57 keV and 40 keV compared to the mixed 120 kVp‐equivalent image.

## 4 DISCUSSION

This study investigated TwinBeam DECT images with the goal of improving the delineation of liver tumors for radiation therapy purposes. Virtual monoenergetic images from TwinBeam dual‐energy data were compared to mixed 120 kVp‐equivalent images by quantifying changes in GTV contrast and CNR for liver tumors. Mixed 120 kVp‐equivalent images created from TwinBeam DECT data represent the baseline GTV contrast and CNR expected from conventional single‐energy CT images.

On average, VMIs at 57 keV increased GTV contrast by 68% compared to mixed 120 kVp‐equivalent images. VMIs at 40 keV increased GTV contrast by 215% compared to mixed 120 kVp‐equivalent images. This is as expected because the attenuation of iodine increases with deceasing energy. Although on average the venous phase demonstrated greater GTV contrast, not all tumor cases followed this trend. Different liver tumors have different enhancement properties,^1^, ^7^ which is illustrated by the fact that some cases showed greater GTV contrast during the arterial phase compared to the venous phase (Figs. 2 and 3). These results support other studies which say that dual‐phase imaging is crucial for liver tumor detection as both phases may aid in tumor visualization.^3^

This is one of the first studies to investigate the conspicuity of entire liver tumors with dual‐energy CT.^3^, ^4^, ^5^, ^6^ The investigation of absolute contrast and CNR of the entire liver GTV rather than just a small ROI is relevant to radiation therapy as the entire tumor needs to be segmented for accurate treatment, and optimally placed ROIs do not represent the detectability of the entire GTV. Other studies have investigated the use of DECT images for liver tumor detection using optimally placed small ROIs and found that lower‐energy images provide greater tumor conspicuity.^3^, ^4^ Robinson et al. investigated the conspicuity of hypovascular liver metastases using 80 kVp images and virtual 120 kVp images from sequential scanning DECT.^6^ Since Robinson et al. used small ROIs placed within the liver lesion rather than the entire GTV to calculate absolute contrast, the resulting values were much higher compared to our study (78.37 ± 24.6 HU for the 80 kVp image and 56.89 ± 17.9 HU for the virtual 120 kVp image compared to 54.9 ± 32.2 HU for the VMIs at 40 keV and 19.5 ± 13.4 HU for mixed 120 kVp‐equivalent images in our study). Sequential scanning DECT has greater spectral separation and lower image noise compared to TwinBeam DECT.^11^ Therefore, the superior DECT technique and the use of optimally placed ROIs are likely factors that resulted in the Robinson et al. study achieving statistically greater CNR values while our study did not. Marin et al. investigated hypervascular liver tumors on 80 kVp and 140 kVp images from sequential scanning DECT. Marin et al. also calculated CNR using a small ROI placed within the liver lesion and found higher CNR values than in our study. Marin et al. CNR values were 6.4 ± 1.0 and 8.2 ± 1.0 for the 140 kVp and 80 kVp images, respectively, which is greater than the mean ± SD GTV CNR of 2.9 ± 1.8 for the VMIs at 40 keV from our study. Marin et al. investigated a total of 83 hypervascular liver tumors and Robinson et al. investigated 44 hypovascular tumors. One limitation of our study is that only 20 tumor cases of varying diagnosis and enhancement properties were investigated. A greater subject population of a single tumor type may improve the statistics of our study. Another limitation of our study is that the mean HU within the GTV and healthy tissue ROI was used rather than median HU. This could have caused our results to be subject to outliers and not truly represent detectability. The use of median HU may improve contrast and CNR values and better represent detectability.

**Fig. 4:**
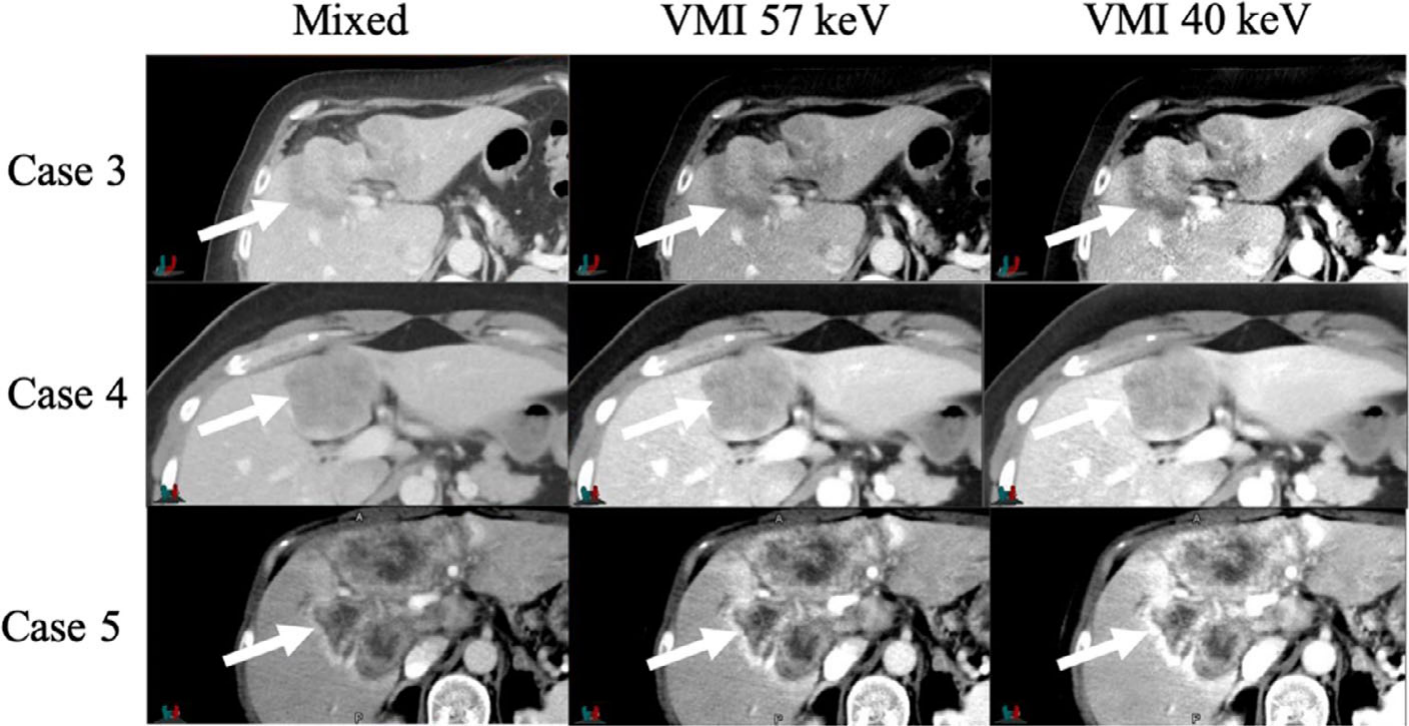
Mixed, VMI at 57 keV, and VMI at 40 keV of heterogeneous tumors during the phase with the greatest GTV contrast. The arrow indicates the location of the GTV

As expected, image noise was the greatest for the VMIs at 40 keV even with the use of ADMIRE at a strength of 2. This agrees well with published data.^10^ Although the GTV contrast was significantly greater in the VMIs than the mixed 120 kVp‐equivalent images, the gain in absolute contrast did not completely overcome the increase in image noise for every case. One potential explanation as to why the low‐energy VMIs on average did not provide statistically greater CNR is that this study investigated entire liver GTVs to calculate CNR rather than small optimally placed ROIs. Liver tumors can be very heterogeneous due to vascular heterogeneity causing regions of hypoxia or regions of greater enhancement. These hypo‐ or hyperintense regions will then get averaged out when considering the GTV as a whole. Figure 4 shows examples of heterogeneous tumors from our study. For these specific cases, the VMIs at 40 keV did not have a greater GTV CNR than the mixed 120 kVp‐equivalent images. We did, however, separate the cohort based on tumors that were not visually heterogeneous, and although the sample size was small, we did see significant improvements in CNR with the VMI at 40 keV for these specific cases. This included both hypo‐ and hyperattenuating tumors.

**Fig. 5:**
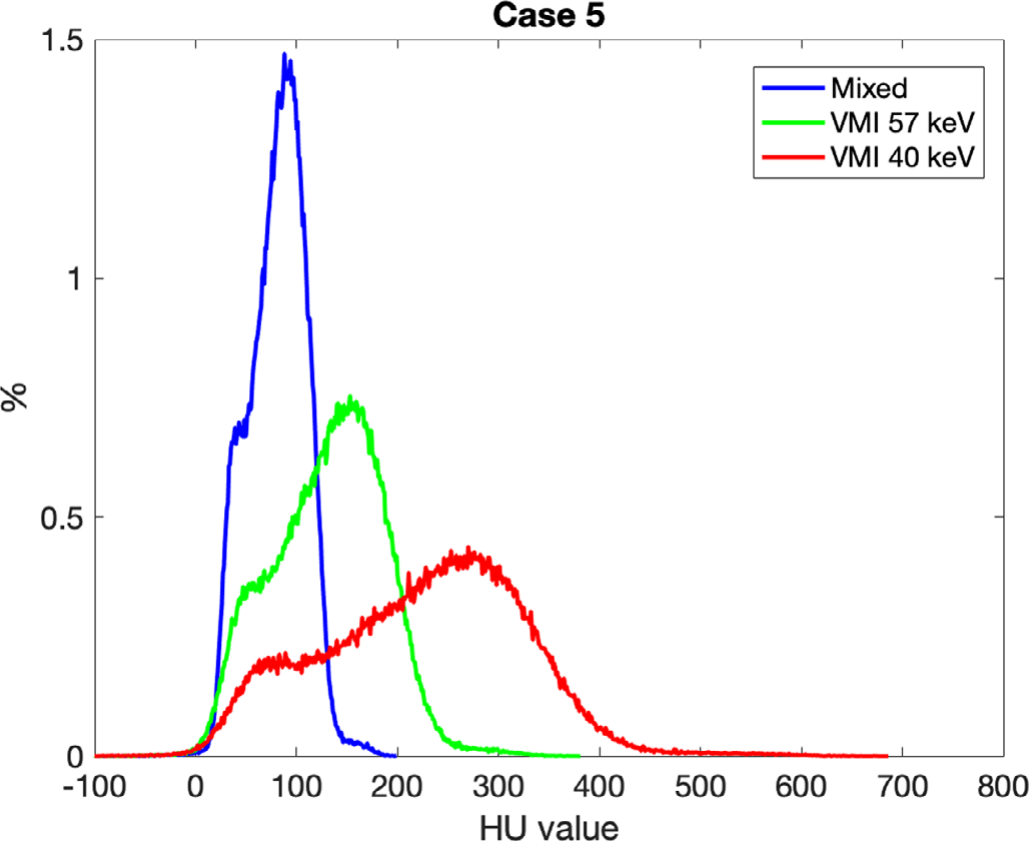
Histograms of the liver GTV from the mixed 120 kVp‐equivalent image, VMI at 57 keV, and VMI at 40 of Case 5 of Fig. 4. The y‐axis is the percent of the total number of pixels within the GTV with that specific HU value with 1 HU bin widths. This graph illustrates the increase in GTV heterogeneity with low‐energy VMIs

The heterogeneity of the GTV and the use of a small ROI were further investigated for Case 5. Figure 5 shows the histograms of the GTV of Case 5. These histograms provide a quantitative depiction of the heterogeneity of the tumor and one can conclude that an ROI placed in a high‐contrast region will provide different CNR values than the average value of the entire GTV. The CNR calculated from the GTV of Case 5 was 1.93 and 0.25 for the mixed 120 kVp‐equivalent and VMI at 40 keV image, respectively. When an optimally placed ROI was used to calculate CNR, the values increased to 8.37 and 9.53 for the mixed and VMI at 40 keV, respectively. This example demonstrates that the VMIs at 40 keV can provide a greater CNR than the mixed 120 kVp‐equivalent image when an optimally placed ROI is used. Therefore, it is hypothesized that if optimally placed ROIs were used to calculate absolute contrast and CNR for all cases similar to previous studies, then the calculated CNR would be significantly greater for TwinBeam low‐energy VMIs. As previously stated, the use of small ROIs to determine tumor conspicuity is not as meaningful for radiation therapy applications.

Overall, TwinBeam is a cost‐effective, single‐source DECT that can be used for dynamic contrast imaging. TwinBeam VMIs at 40 keV demonstrated greater contrast of liver tumors, and for some cases, these images provided greater CNR than conventional single‐energy CT images (Figs. 2 and 3). This was not true for all cases investigated, which is why on average there was not a statistically significant increase in CNR for low‐energy VMIs. The tumors investigated for this study were very heterogeneous, so a texture analysis study of these cases is suggested, as results may lead to other methods of using TwinBeam DECT for radiation therapy purposes. Texture analysis may reveal that for cases where TwinBeam DECT imaging does not improve CNR, it may improve the edge detection for liver tumors or enhance other imaging features that may aid in tumor delineation. A contouring study investigating the reproducibility and accuracy of GTV contours is also suggested as it may determine other clinical implications TwinBeam DECT images have in the field of radiation therapy. Results from a contouring study may also determine if the quantitative increase in absolute contrast correlates with improvements in lesion detectability and a decrease in contour variability.

## CONFLICT OF INTERESTS

This work was partially funded by a collaboration with Siemens Healthineers.
